# Relationships of coronary culprit-plaque characteristics with duration of diabetes mellitus in acute myocardial infarction: an intravascular optical coherence tomography study

**DOI:** 10.1186/s12933-019-0944-8

**Published:** 2019-10-19

**Authors:** Zhaoxue Sheng, Peng Zhou, Chen Liu, Jiannan Li, Runzhen Chen, Jinying Zhou, Li Song, Hanjun Zhao, Hongbing Yan

**Affiliations:** Department of Cardiology, Fuwai Hospital, National Center for Cardiovascular Diseases, Peking Union Medical College & Chinese Academy of Medical Sciences, No.167, Beilishi Road, Xicheng District, Beijing, 100037 China

**Keywords:** Acute myocardial infarction, Diabetes mellites, Diabetes duration, Optical coherence tomography, TCFA

## Abstract

**Background:**

Diabetes mellitus (DM) or pre-diabetes status is closely associated with features of vulnerable coronary lesions in patients with stable coronary heart disease or acute coronary syndrome. However, the association between duration of diabetes and the morphologies and features of vulnerable plaques has not been fully investigated in patients with acute myocardial infarction (AMI).

**Methods:**

We enrolled a total of 279 patients who presented with AMI between March 2017 and March 2019 and underwent pre-intervention optical coherence tomography imaging of culprit lesions. Patients with DM were divided into two subgroups: a Short-DM group with DM duration of < 10 years and a Long-DM group with DM duration of ≥ 10 years. Baseline clinical data and culprit-plaque characteristics were compared between patients without DM (the non-DM group), those in the Short-DM group, and those in the Long-DM group.

**Results:**

Patients with DM represented 34.1% of the study population (95 patients). The Short- and Long-DM groups included 64 (67.4%) and 31 patients (32.6%), respectively. Glycated hemoglobin A1c (HbA1c) levels were significantly higher in the Long-DM group than the Non- or Short-DM groups (8.4% [Long-DM] versus 5.7% [Non-DM] and 7.6% [Short-DM], P < 0.001). In addition, the highest prevalence of lipid-rich plaques, thin-cap fibroatheroma (TCFA), and plaque ruptures of culprit lesions were observed in the Long-DM group (lipid-rich plaques: 80.6% [Long-DM] versus 52.2% [Non-DM] and 62.5% [Short-DM], P = 0.007; TCFA: 41.9% [Long-DM] versus 19.6% [Non-DM] and 31.3% [Short-DM], P = 0.012; plaque rupture: 74.2% [Long-DM] versus 46.7% [Non-DM] and 48.4% [Short-DM], P = 0.017). The frequency of calcification was significantly higher among patients with DM than among those without (62.1% versus 46.2%, P = 0.016); however, no significant differences were found between the DM subgroups (61.3% [Long-DM] versus 62.5% [Short-DM], P = 0.999).

**Conclusions:**

Increased duration of DM combined with higher HbA1c levels influences culprit-plaque characteristics in patients with DM who suffer AMI. These findings might account for the higher risks of cardiac death in DM patients with long disease duration.

*Trial registration* This study is registered at clinicaltrials.gov as NCT03593928

## Background

Patients with diabetes mellitus (DM) are at an increased risk of cardiovascular morbidity and mortality [1]. The duration of DM has a significant influence on the cause of death; increased duration of disease is associated with increased risk of coronary heart disease mortality [2]. Optical coherence tomography (OCT) is a high-resolution, intravascular imaging modality that enables detailed assessment of the characteristics of coronary plaques [3, 4]. Previous studies have reported that type 2 DM (T2DM) or pre-diabetes can have enormous impacts on the characteristics of coronary atherosclerotic plaques, as assessed by OCT, in patients with stable coronary artery disease (CAD) or acute coronary syndrome (ACS) [5–8]. However, the association between duration of DM and the morphology and features of coronary culprit vulnerable plaques has not been fully investigated in patients with acute myocardial infarction (AMI) using OCT. Therefore, the present study aimed to explore these characteristics in relation to duration of DM in patients who presented with AMI, to elucidate the effects of duration of DM on the incidence of acute coronary ischemic events.

## Methods

### Study population

We recruited consecutive patients aged ≥ 18 years who presented with ST-segment elevation myocardial infarction (STEMI) and underwent primary percutaneous coronary intervention at Fuwai Hospital between March 2017 and March 2019. All patients were screened for OCT examination (Fuwai Hospital OCTAMI Registry, clinical trials.gov: NCT03593928). The main exclusion criteria were: cardiogenic shock, end-stage renal disease, serious liver dysfunction, allergy to contrast media, and contraindication to aspirin or ticagrelor. Furthermore, patients with left main coronary artery disease or extremely tortuous or heavily calcified vessels were excluded because of potential difficulties in performing OCT. We defined STEMI as continuous chest pain lasting > 30 min, ST-segment elevation of > 0.1 mV in at least two contiguous leads, or new left bundle-branch block on the 18-lead electrocardiogram and elevated troponin I level [9].

Diagnosis of T2DM was based on clinical records and was made according to the American Diabetes Association criteria of glycated hemoglobin (HbA1c) level of ≥ 6.5% and fasting glucose level of ≥ 126 mg/dL or 2-h plasma glucose level of ≥ 200 mg/dL during the oral glucose tolerance test as well as use of anti-diabetic drugs [10]. Duration of DM was calculated as the time period from the first diagnosis or the first claimed prescription of glucose-lowering agents, whichever was earlier, until the time of admission for STEMI. Patients with DM were further divided into two subgroups: patients with a short duration of DM (denoted the Short-DM group, DM duration < 10 years) and those with a long duration of DM (Long-DM group, DM duration ≥ 10 years). Baseline clinical parameters and culprit-plaque characteristics were compared between the patients without DM (the Non-DM group), Short-DM, and Long-DM groups.

### Procedural data and culprit lesion identification

Coronary angiography was performed via the transradial or transfemoral approach with a 6F or 7F sheath. All patients received standard-of-care therapy according to international guidelines [9]; namely, initial oral treatment with 300 mg of aspirin followed by 75–100 mg daily, initial administration of 180 mg ticagrelor followed by 90 mg twice daily for ≥ 12 months or 600 mg clopidogrel followed by 75 mg daily for ≥ 12 months, and intravascular infusions of 70–100 IU/kg of unfractionated heparin prior to percutaneous coronary intervention. Infusions of glycoprotein IIb/IIIa receptor inhibitors were administered if necessary.

The culprit vessel was determined primarily by coronary angiography and corroborated with electrocardiogram information and regional-wall motion abnormalities from echocardiographic or ventriculographic assessments.

### Optical coherence tomography image acquisition

We performed intravascular OCT imaging as previously described [3]. Briefly, OCT images of the culprit lesions were acquired using the frequency-domain OCT system (ILUMIEN OPTIS™, St. Jude Medical/Abbott, St. Paul, MN, USA) and a dragonfly catheter (Lightlab Imaging, Inc., Westford, MA, USA) after restoration of antegrade blood flow with thrombus aspiration and/or gentle predilatation. During image acquisition, coronary blood flow was displaced by continuously flushing with contrast media via manual injection directly from the guiding catheter, to create a virtually blood-free environment. Images were acquired with an automated pullback at a rate of 36 mm/s, and cross-sectional images were generated at a rotational speed of 180 frames/s. The total length of OCT pullback was 75 mm.

### Optical coherence tomography image analysis

All OCT images were analyzed on a St. Jude OCT Offline Review Workstation by three independent investigators who were blinded to angiographic and clinical data. The first investigator was primarily responsible for screening suitability for culprit-plaque evaluation. The other two investigators analyzed OCT images. Disagreements were resolved by consensus. Optical coherence tomography analysis was conducted along the entire OCT pullback in order to determine entire segments of culprit plaques. The culprit plaque was defined as the segments centered on the culprit lesion and extending bilaterally to ≥ 5 mm of normal vessel segment [11].

**Fig. 1:**
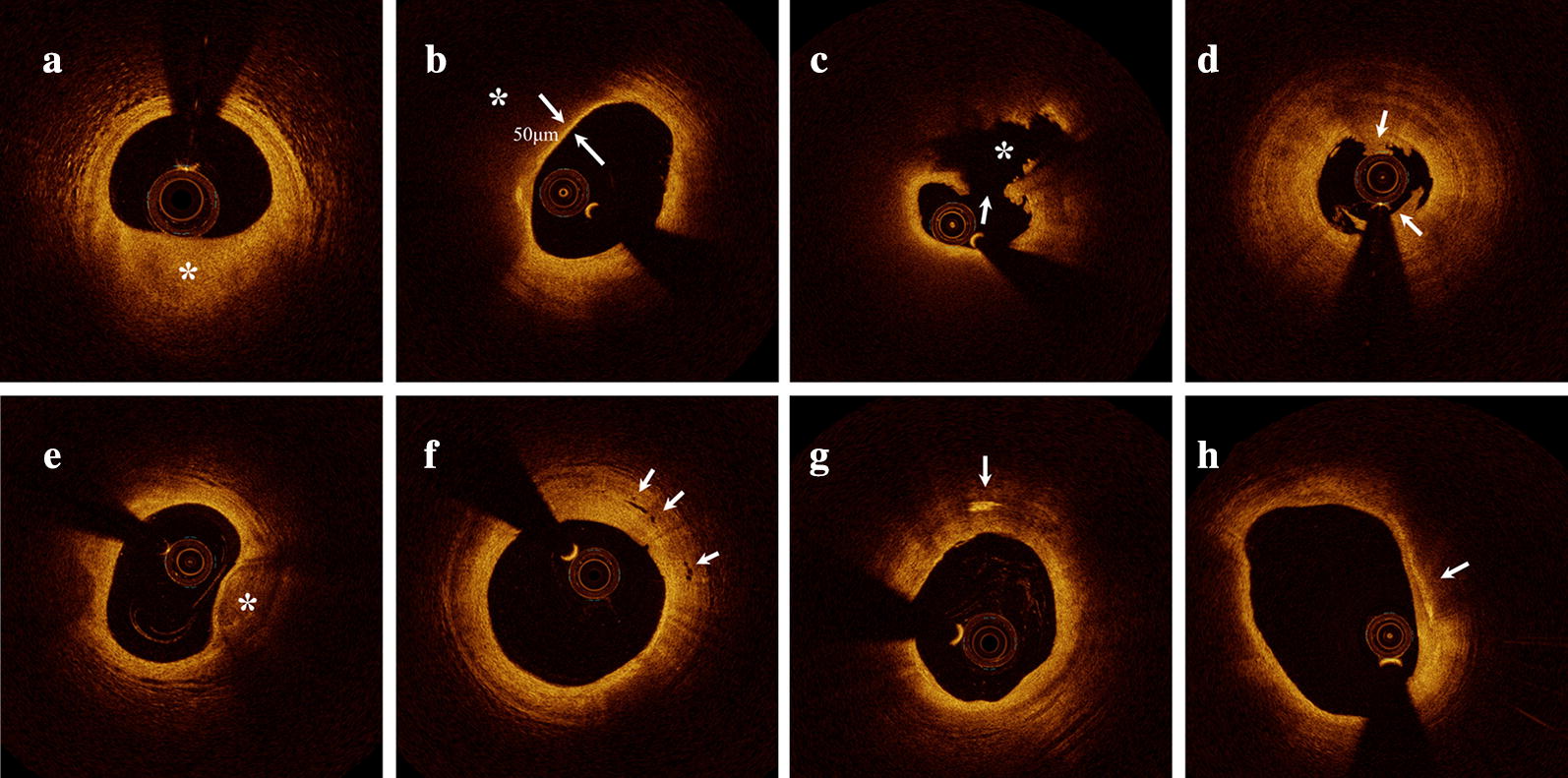
Representative cross-sectional optical coherence tomography images. **a** Fibrous plaque identified as a homogeneous, highly backscattering region (asterisk). **b** Lipid-rich plaque identified as a low-signal region with a diffuse border (asterisk) and thin-cap fibroatheroma with fibrous-cap thickness of 50 μm. **c** Plaque rupture identified by disruption of the fibrous cap (arrow) and cavity formation (asterisk). **d** Plaque erosion identified by the presence of attached thrombus (arrow) overlying an intact plaque. **e** Calcification identified by the presence of a well-delineated, low-backscattering heterogeneous region (asterisk). **f** Microvessels defined as tubule luminal structures that do not generate a signal, with no connection to the vessel lumen (arrow). **g** Cholesterol crystal (arrow) identified by linear, highly backscattering structures without remarkable backward shadowing. **h** Macrophage infiltration (arrow) defined as a signal-rich, distinct or confluent punctate region of higher intensity than background speckle noise that generates remarkable backward shadowing

According to established criteria [3], culprit plaques were classified as fibrous or lipid-rich plaques, identified as a homogeneous, highly backscattering region (Fig. 1a) or a low-signal region with a diffuse border (Fig. 1b), respectively. Plaque rupture was identified by disruption of the fibrous cap with clear cavity formation (Fig. 1c). Plaque erosion was defined by OCT evidence of thrombus, an irregular luminal surface, and no evidence of cap rupture in multiple adjacent frames (Fig. 1d). Thin-cap fibroatheroma (TCFA) was defined as a lipid-rich plaque (maximum lipid arc greater than two quadrants), with the thinnest part of the fibrous cap being < 65 μm (Fig. 1b). The lipid arc was measured at 1-mm intervals across the entire lesion, and the largest arc recorded. Fibrous-cap thickness was measured in triplicate at the thinnest part of the fibrous cap of the culprit plaque, and the average value calculated. The length of the culprit lesion was measured as the span of the entire culprit plaque in the longitudinal view.

Calcification within plaques was identified by the presence of well-delineated, low-backscattering heterogeneous regions (Fig. 1e). Microvessels were defined as tubule luminal structures without a connection to the vessel lumen that did not produce a signal, recognized in more than three consecutive cross-sectional OCT images (Fig. 1f). Cholesterol crystals were defined as linear, highly backscattering structures within the plaque (Fig. 1g). Macrophage infiltration was defined as signal-rich, distinct or confluent punctate regions above the intensity of background speckle noise with backward shadowing, usually located at the boundary between the fibrous cap and inner lipid core (Fig. 1h). Thrombus was defined as an irregular mass protruding into the lumen or adjacent to the luminal surface. The minimal lumen area (MLA) was evaluated along the length of the target lesion.

### Statistical analysis

Continuous data are presented as mean ± standard deviation (SD) or median (25th, 75th percentiles). Between-group differences were analyzed using one-way analysis of variance (ANOVA). Categorical data are presented as number (%), and were compared using Pearson’s χ^2^ or Fisher’s exact test. Further multiple comparison analysis was performed to analyze within-group differences; the post hoc Scheffe method was chosen for continuous data and the Bonferroni method for categorical data. Because the number of patients with DM was relatively small, post hoc power analysis was performed to estimate the reliability of comparisons between the Short- and Long-DM group. Logistic regression analyses with adjustments for confounding factors were used to determine associations of the duration of DM with vulnerable-plaque features determined by OCT. A two-tailed P value of < 0.05 was considered statistically significant.

## Results

### Baseline characteristics

**Fig. 2:**
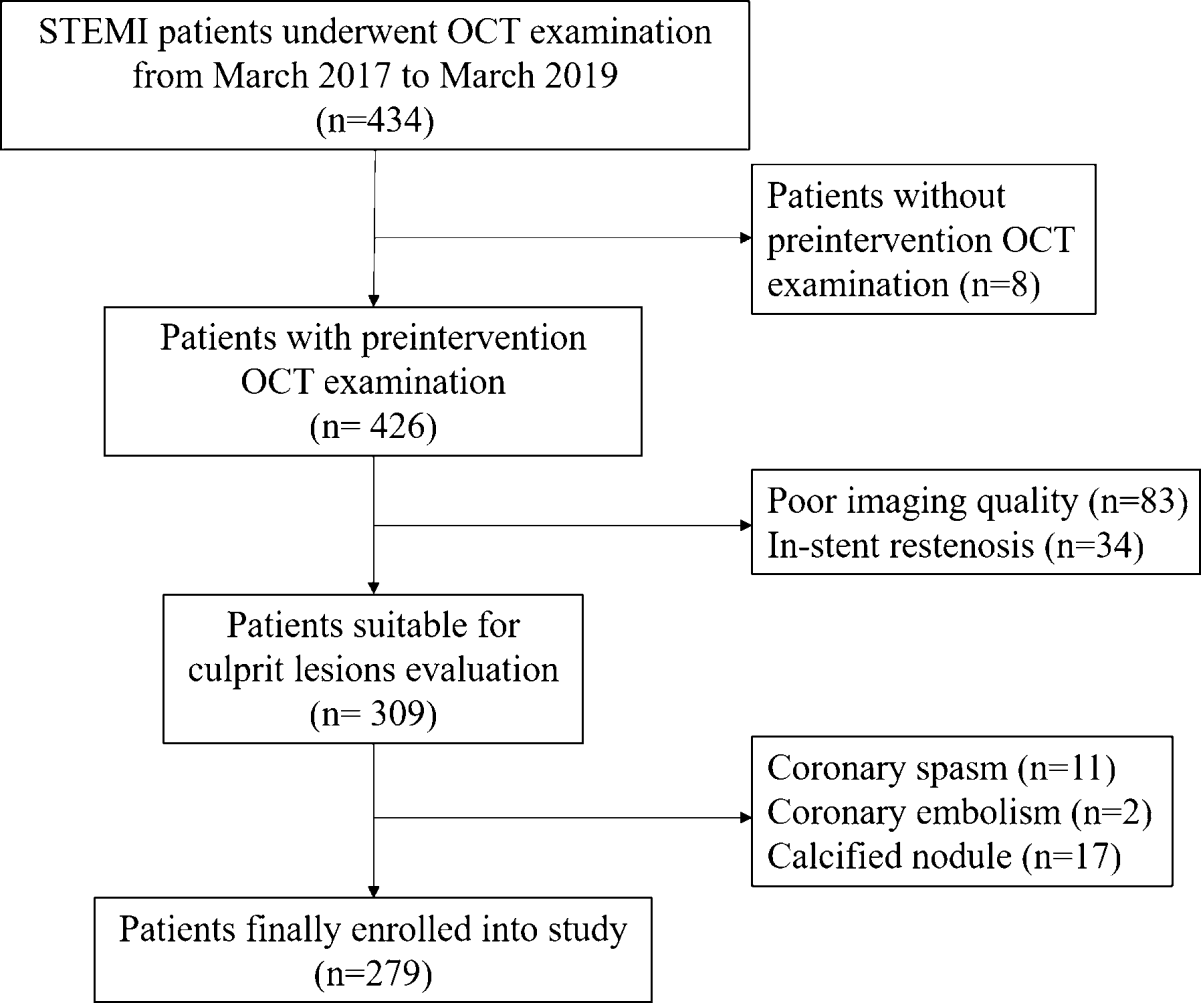
Study flow chart. *OCT* optical coherence tomography, *STEMI* ST-segment elevation myocardial infarction

Between March 2017 and March 2019, we recruited 434 patients who presented with STEMI and underwent OCT imaging of native culprit vessels. Of those, 155 were excluded because of lack of preintervention OCT examinations (n = 8), poor imaging quality due to massive thrombus (n = 83), in-stent restenosis (n = 34), coronary spasm (n = 1), coronary embolism (n = 2), and calcified nodule (n = 17). Finally, 279 patients were enrolled in this study. The study flow chart is displayed in Fig. 2. Comparisons of baseline characteristics between included and excluded patients are detailed in Additional file 1: Table S1. Briefly, included patients had higher levels of total cholesterol (TC) and low-density lipoprotein cholesterol (LDL-C) than excluded patients. However, age; sex; body mass index (BMI); history of hypertension, diabetes, dyslipidemia, and smoking; and other laboratory parameters were not significantly different (Additional file 1: Table S1).

Of the enrolled patients, 95 (34.1%; 77 men and 18 women) were diagnosed with T2DM. Their mean age was 57.1 years and median duration of diabetes was 7.0 (3.0–12.3) years. Table 1 presents a comparison of baseline clinical characteristics between the Non-DM, Short-DM, and Long-DM groups. There were no significant differences in age, sex, BMI, medical history, prior medication, or laboratory findings except for HbA1c and triglyceride levels which were significantly higher in the Short-DM and Long-DM groups than the Non-DM group (P < 0.001 and P = 0.001, respectively). Further multiple comparisons revealed that the Long-DM group had significantly higher levels of HbA1c than the Short-DM group, but TG values were not significantly different (Table 1).

**Table 1:** Baseline clinical characteristics of the study population

Variable	Non-DM (n = 184)	Short-DM (n = 64)	Long-DM (n = 31)	P value	P_Non vs. Short_	P_Short vs. Long_	P_Non vs. Long_
Age, years	57.5 ± 11.6	55.2 ± 11.5	61.0 ± 10.5	0.063			
BMI, kg/m^2^	25.8 ± 4.0	27.1 ± 3.4	26.1 ± 2.8	0.072			
Men	152 (82.6)	50 (78.1)	27 (87.1)	0.586			
Smoking	127 (69.0)	43 (67.2)	24 (77.4)	0.623			
Medical history							
Hypertension	102 (55.4)	41 (64.1)	20 (64.5)	0.378			
Dyslipidemia	168 (91.3)	56 (87.5)	29 (93.5)	0.630			
Prior PCI	12 (6.5)	5 (7.8)	4 (12.9)	0.388			
Laboratory findings							
White blood cell count, × 10^6^/L	10.6 ± 3.2	10.9 ± 3.2	10.0 ± 2.7	0.624			
Hs-CRP, mg/L	5.3 (2.4, 10.9)	6.6 (2.9, 10.3)	4.0 (2.6, 10.6)	0.840			
eGFR, mL/min/1.73 m^2^	97.3 (77.7, 114.3)	99.9 (88.2, 119.1)	93.2 (77.8, 107.3)	0.119			
HbA1c, %	5.7 (5.5, 5.9)	7.6 (6.8, 8.9)	8.4 (7.7, 9.8)	< 0.001*	< 0.001*	0.001*	< 0.001*
TC, mg/dL	164.0 (139.2, 195.2)	172.1 (155.8, 203.2)	175.9 (130.7, 205.0)	0.172			
TG, mg/dL	114.7 (73.7, 167.8)	154.5 (104, 215.8)	144.3 (90.3, 206.3)	0.001*	0.006*	0.996	0.046
LDL-C, mg/dL	104 (82.6, 127.5)	114.5 (95.3, 133.0)	103.6 (78.9,128.4)	0.279			
HDL-C, mg/dL	41.4 (35.6, 48.3)	39.3 (35.3, 45.7)	39.8 (34.8, 47.6)	0.417			
Lipoprotein (a), mg/L	179.8 (84.5, 393)	107.5 (49,311.4)	149.0 (87.1, 315.0)	0.825			
LVEF, %	54.7 ± 6.5	56.6 ± 5.2	53.9 ± 5.4	0.062			
Prior medications							
Aspirin	64 (34.8)	29 (45.3)	12 (38.7)	0.310			
P2Y12 inhibitor	43 (23.4)	17 (26.6)	11 (35.5)	0.323			
Statin	34 (18.5)	8 (12.5)	5 (16.1)	0.592			
DM control							
Insulin		5 (7.8)	11 (35.5)				
Oral hypoglycemic agents		40 (62.5)	18 (58.1)				
Diet only		19 (29.7)	2 (6.5)				

Continuous data are presented as mean ± standard deviation or median (25th, 75th percentile). Categorical data are presented as number (%)

*BMI* body mass index, *DM* diabetes mellitus, *eGFR* estimated glomerular filtration rate, *HbA1c* glycosylated hemoglobin, *HDL*-*C* high-density lipoprotein cholesterol, *Hs*-*CRP* high-sensitivity C-reactive protein, *PCI* percutaneous coronary intervention, *LDL*-*C* low-density lipoprotein cholesterol, *Long*-*DM* patients with a long duration of diabetes, *LVEF* left ventricular ejection fraction, *Non*-*DM* patients without diabetes, *Short*-*DM* patients with a short duration of diabetes, *TC* total cholesterol, *TG* triglyceride

* P < 0.05

### Procedure data

Procedure data are shown in Table 2. Briefly, there were no differences among the three groups in terms of distribution of culprit vessels or incidence of pre-dilation, aspiration, or pre-intervention TIMI grade flow of ≤ 1. Notably, the difference in the number of diseased vessels was borderline significant between groups (P = 0.099), and the frequency of multiple-vessel diseases was higher among DM patients than those without DM, especially compared with the Long-DM group (P = 0.043).

**Table 2:** Procedural data

Variables	Non-DM (n = 184)	Short-DM (n = 64)	Long-DM (n = 31)	P value
Angiographic findings				
Culprit vessels				0.663
LAD	88 (47.8)	31 (48.4)	16 (51.6)	
LCX	23 (12.5)	4 (6.3)	2 (6.5)	
RCA	73 (39.7)	29 (45.3)	13 (41.9)	
Coronary artery lesions				0.099
SVD	55 (30.7)	15 (23.4)	3 (10.0)	
DVD	62 (34.6)	20 (31.3)	12 (40.0)	
TVD	62 (34.6)	29 (45.3)	15 (50.0)	
LM disease	5 (2.7)	0	1 (3.2)	0.428
Multivessel disease	124 (69.3)	49 (76.6)	27 (90.0)	0.043*
Procedures				
Aspiration	150 (81.5)	48 (75.0)	24 (77.4)	0.484
Pre-dilation	108 (58.7)	45 (70.3)	20 (64.5)	0.249
Pre-TIMI flow ≤ 1	135 (73.4)	41 (64.1)	20 (64.5)	0.268

Data are presented as number (%)

*DVD* double vessel disease, *LAD* left anterior descending artery, *LCX* left circumflex artery, *LM* left main coronary artery, *Long*-*DM* patients with a long duration of diabetes, *Non*-*DM* patients without diabetes, *RCA* right coronary artery, *Short*-*DM* patients with a short duration of diabetes, *SVD* single vessel disease, *TVD* triple vessel disease

* P < 0.05

### Optical coherence tomography findings

**Fig. 3:**
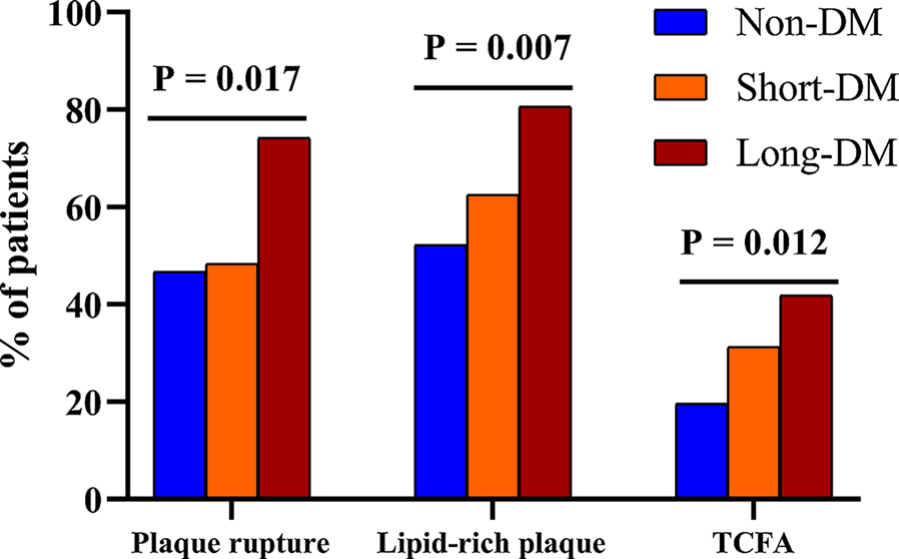
Bar graphs of optical coherence tomography findings of coronary plaques between groups. Comparisons of the incidence of plaque rupture, lipid-rich plaques, and thin-cap fibroatheroma showed significant differences between patients in the Non-DM, Short-DM (< 10 years duration of disease), and Long-DM (≥ 10 years duration of disease) groups. *DM* diabetes mellitus, *TCFA* thin-cap fibroatheroma

Representative OCT images are shown in Fig. 1. Comparisons of OCT findings between the three groups are shown in Table 3. The percentage of lipid-rich plaques in culprit lesions was higher among patients with DM than those without (68.4% versus 52.2%, P = 0.011); further multiple comparisons showed that lipid-rich plaques were more prevalent in the Long-DM and Short-DM groups than the Non-DM group, but this difference was only significant between the Long-DM and Non-DM groups (Fig. 3). Similarly, the prevalence of TCFA was higher in patients with DM than those without (34.7% versus 19.6%, P = 0.008). Further multiple comparisons revealed that the difference was only significant between the Long-DM and Non-DM groups (41.9% versus 19.6%, P = 0.010), with no significant differences between the DM subgroups (41.9% versus 31.3%, P = 0.361) with a power value of 0.177 (Fig. 3).

**Table 3:** Optical coherence tomography characteristics

Variables	Non-DM (n = 184)	Short-DM (n = 64)	Long-DM (n = 31)	P value				
				P_for overall_^**#**^	P_Non-DM vs. DM_	P_Non-DM vs. Short-DM_	P_Short-DM vs. Long-DM_	P_Non-DM vs. Long-DM_
Plaque morphology				0.017*	0.130	0.885	0.026	0.006*
Plaque rupture	86 (46.7)	31 (48.4)	23 (74.2)					
Intact fibrous cap	98 (53.3)	33 (51.6)	8 (25.8)					
Plaque type				0.007*	0.011*	0.189	0.100	0.003*
Lipid-rich plaque	96 (52.2)	40 (62.5)	25 (80.6)					
Fibrous plaque	88 (47.8)	24 (37.5)	6 (19.4)					
TCFA	36 (19.6)	20 (31.3)	13 (41.9)	0.012*	0.008*	0.059	0.361	0.010*
Calcification	85 (46.2)	40 (62.5)	19 (61.3)	0.043*	0.016*	0.029	1.000	0.126
Macrophage	95 (51.6)	38 (59.4)	19 (61.3)	0.407	0.206	0.311	1.000	0.338
Microvessels	32 (17.4)	12 (18.8)	5 (16.1)	0.970	1.000	0.850	0.787	1.000
Cholesterol crystal	10 (5.4)	8 (12.5)	4 (12.9)	0.080	0.058	0.089	1.000	0.124
Thrombus	178 (96.7)	63 (98.4)	31 (100)	0.725	0.429	0.681	1.000	0.597
Minimal FCT, μm	123 ± 84	125 ± 97	101 ± 73	0.387	0.065	0.993	0.456	0.416
Stenosis length, mm	18.0 ± 5.8	18.6 ± 7.1	20.9 ± 7.2	0.058	0.092	0.814	0.240	0.059
Maximal lipid arc, °	300 ± 75	301 ± 71	333 ± 47	0.059	0.255	0.999	0.123	0.064
MLA, mm^2^	1.88 ± 0.75	1.89 ± 0.66	1.90 ± 0.64	0.975	0.830	0.989	0.997	0.981

Continuous data are presented as mean ± standard deviation. Categorical variables are presented as number (%)

*DM* diabetes mellite*s, FCT* fibrous cap thickness, *Long*-*DM* patients with a long duration of diabetes, *MLA* minimal lumen area, *Non*-*DM* patients without diabetes, *Short*-*DM* patients with a short duration of diabetes, *TCFA* thin-cap fibroatheroma

* P < 0.05

^**#**^P_for overall_ means statistical analysis among three groups

The frequency of plaque rupture was comparable between the Non-DM group and patients with DM (46.7% versus 56.8%, P = 0.130). Multiple comparisons within groups revealed a significantly higher incidence of plaque rupture in the Long-DM group compared with the Non-DM group (74.2% versus 46.7%, P = 0.006), although a statistically significant difference was not observed between the Long- and Short-DM groups (74.2% versus 48.4%, P > 0.05), with a power value of 0.675 (Fig. 3).

The frequencies of microstructural features including macrophage infiltration, microvessels, cholesterol crystal, and thrombus were similar in all three groups, as were quantitative parameters including stenosis length, maximum lipid arc, fibrous-cap thickness, and MLA. However, the prevalence of calcification was higher among patients with DM than those without (62.1% versus 46.2%, P = 0.016). No significant differences were found between the subgroups.

Univariate logistic regression analysis showed that duration of DM was closely associated with presence of plaque rupture (Table 4) and TCFA (Table 5). After adjustment for potential confounding factors including age, sex, BMI, hypertension, smoking, total cholesterol, triglyceride, LDL-C, high-density lipoprotein cholesterol (HDL-C), high-sensitivity C-reactive protein (hs-CRP), estimated glomerular filtration rate (eGFR), and prior statin therapy, duration of DM remained predictive for plaque rupture and TCFA (Tables 4, 5).

**Table 4:** Logistic regression analysis of plaque rupture

Variables	Univariate		Multivariate	
	OR (95% CI)	P value	OR (95% CI)	P value
Age, years	1.03 (1.01–1.05)	0.003*	1.03 (1.01–1.05)	0.017*
Men	1.49 (0.81–2.77)	0.203		
BMI, kg/m^2^	1.00 (0.94–1.07)	0.910		
Diabetes group				
Short vs. Non	1.07 (0.61–1.90)	0.815	1.23 (0.68–2.20)	0.495
Long vs. Non	3.28 (1.39–7.70)	0.007*	2.96 (1.24–7.05)	0.014*
Hypertension	1.01 (0.63–1.63)	0.960		
Smoking	0.85 (0.51–1.42)	0.542		
TC, mg/dL	1.00 (0.99–1.01)	0.742		
TG, mg/dL	1.00 (1.00–1.00)	0.598		
LDL-C, mg/dL	1.00 (0.99–1.01)	0.729		
HDL-C, mg/dL	1.00 (0.98–1.02)	0.894		
Hs-CRP, mg/L	0.98 (0.93–1.03)	0.392		
eGFR, mL/min/1.73 m^2^	0.99 (0.98–0.99)	0.007*	0.99 (0.98–0.99)	0.031*
Prior statin use	1.04 (0.56–1.95)	0.894		

*BMI* body mass index, CI, confidence interval, *HDL*-*C* high-density lipoprotein cholesterol, *eGFR* estimated glomerular filtration rate, *Hs*-*CRP* high-sensitivity C-reactive protein, *LDL*-*C* low-density lipoprotein cholesterol, *Long*-*DM* patients with a long duration of diabetes, *Non*-*DM* patients without diabetes, OR, odds ratio, *Short*-*DM* patients with a short duration of diabetes, *TC* total cholesterol, *TG* triglyceride

* P < 0.05

**Table 5:** Logistic regression analysis of thin-cap fibroatheroma

Variables	Univariate		Multivariate	
	OR (95% CI)	P value	OR (95% CI)	P value
Age, years	1.02 (0.99–1.05)	0.119		
Men	1.62 (0.74–3.53)	0.227		
BMI, kg/m^2^	0.95 (0.87–1.03)	0.207		
Diabetes group				
Short vs. Non	1.87 (0.98–3.55)	0.056	1.78 (0.93–3.40)	0.082
Long vs. Non	2.97 (1.33–6.61)	0.008*	3.06 (1.36–6.88)	0.007*
Hypertension	0.66 (0.38–1.14)	0.136		
Smoking	1.33 (0.72–2.45)	0.363		
TC, mg/dL	1.01 (1.00–1.01)	0.072		
TG, mg/dL	1.00 (1.00–1.00)	0.123		
LDL-C, mg/dL	1.01 (1.00–1.02)	0.043*	1.01 (1.00–1.02)	0.048*
HDL-C, mg/dL	0.99 (0.97–1.02)	0.736		
Hs-CRP, mg/L	0.96 (0.91–1.02)	0.261		
eGFR, mL/min/1.73 m^2^	0.99 (0.98–1.00)	0.063		
Prior statin use	0.58 (0.26–1.30)	0.183		

*BMI* body mass index, CI, confidence interval, *HDL*-*C* high-density lipoprotein cholesterol, *eGFR* estimated glomerular filtration rate, *Hs*-*CRP* high-sensitivity C-reactive protein, *LDL*-*C* low-density lipoprotein cholesterol, *Long*-*DM* patients with a long duration of diabetes, *Non*-*DM* patients without diabetes, OR, odds ratio, *Short*-*DM* patients with a short duration of diabetes, *TC* total cholesterol, *TG* triglyceride

* P < 0.05

## Discussion

To the best of our knowledge, this is the first study to explore the association of duration of DM with morphological characteristics of culprit plaques determined by OCT in STEMI patients. Our study has two major findings: (1) patients with duration of DM of ≥ 10 years have higher plasma HbA1c levels than those with disease duration of < 10 years and (2) patients with DM who present with STEMI, especially those with disease durations of ≥ 10 years, have a higher prevalence of lipid-rich plaques, TCFA, and plaque rupture than those without DM.

It is well known that HbA1c levels reflect glycemic control over a 2- to 3-month period; levels of < 7% are considered optimal. Our study reveals that patients who have suffered from DM for ≥ 10 years have higher plasma HbA1c levels than those without DM or than patients with a disease duration of < 10 years. These data support the findings of previous studies that report increasing duration of diabetes to result in progressively higher HbA1c levels [12, 13]. Together, these results imply that increased duration of diabetes is associated with poorer glycemic control.

### Lipid-rich plaques and thin-cap fibroatheroma

Previous studies have identified thin fibrous-cap thickness (< 65 μm) overlying a large lipid-rich plaque (namely, TCFA) with increased macrophage infiltration and calcification deposition to be pivotal precursors for plaque rupture and subsequent acute coronary ischemic events [14]. The technique of OCT is a unique in vivo intravascular imaging modality which allows visualization of microstructures such as calcification deposits, macrophage accumulation, microvessels, and cholesterol crystals [3]. A pathologic study has demonstrated that, compared with those without DM, patients with DM had plaques with larger necrotic cores and increased macrophage infiltration, and that the size of the necrotic core was positively correlated with HbA1c level [15]. However, previous serial OCT studies have reported inconsistent findings regarding the prevalence of lipid-rich plaques and TCFA between patients with and without DM, as well discrepancies in quantitative parameters of lipid-rich plaques such as minimal fibrous-cap thickness, plaque length, lipid arc, and lipid index [5, 6, 8, 16–19]. Our study demonstrates that DM is associated with a significantly higher incidence of lipid-rich plaques and TCFA, but that quantitative characteristics of plaques are similar between patients with and without DM. In support of these findings, the study of Sugiyama et al. [6], which included 322 patients with ACS, reported the prevalence of lipid-rich plaques to be higher among patients with DM compared with those without (58.9% versus 44.9%, P = 0.030). Another study by De Rosa et al. [16], which included 67 stable patients with CAD, reported the incidence of fibroatheroma (P = 0.015) and TCFA (P = 0.011) to be higher among patients with DM. Notably, in the present study, multiple comparison of patients with long durations of DM with those without DM revealed the differences in the frequencies of lipid-rich plaque and TCFA to be more significant. This implies that long-term exposure to hyperglycemia, together with deteriorating insulin resistance, results in advanced atherosclerosis and high levels of systemic and local inflammation, thereby promoting the progression of coronary atherosclerosis [20, 21]. Moreover, multivariate logistics analysis indicated that, as well as longer duration of DM, higher LDL-C levels were associated with the incidence of TCFA. This is consistent with previous studies that reported diabetes status, as well as plasma LDL-C, to significantly influence plaque characteristics [22, 23].

### Plaque rupture

Driven by multiple pathophysiological disturbances, patients with DM are predisposed to a proinflammatory prothrombotic state, which may lead to plaque rupture [24]. In contrast, a postmortem study of 438 patients who suffered sudden death failed to show differences in the incidence of plaque rupture between patients with (n = 101) and without DM (n = 337) [25]. In addition, recent OCT studies have reported the prevalence of plaque rupture in both culprit [6] and non-culprit lesions [5] to be similar between patients with and without DM. Consistent with these findings, the present study did not identify a significant difference in the frequency of plaque rupture between the two main groups. Interestingly, the proportion of plaque rupture was higher in the Long-DM group than in the Non-DM group. This is a novel finding which implies that duration of DM might play a specific role in the occurrence of plaque rupture and subsequent AMI. Increased coronary atherosclerotic plaque burden and longer duration of exposure to diabetes-related inflammatory factors resulting in a more active prothrombogenic state and reduced response to antithrombotic therapeutic approaches may explain the increased prevalence of plaque rupture that we observed among patients with long disease durations [26, 27]. Notably, differences in the prevalence of TCFA and plaque rupture were not statistically significant between the Long- and Short-DM groups, which may be partly attributable to the relatively small study population, considering the low power values (0.177 and 0.675, respectively). Further investigations involving larger study populations are warranted to clarify this issue.

It also should be noted that the prevalence of plaque rupture (50.2%) of STEMI patients in the present study was much lower than that (70%) reported in a meta-analysis by Iannaccone et al. [28]. This discrepancy may be due to differences in population size and selection. For example, patients with poor cardiac function or severe coronary disease did not undergo OCT examination for ethical and safety reasons in the present study, and 83 patients with high residual thrombus burden were excluded from our final analysis. These exclusions undoubtedly had an impact on the proportion of plaque rupture that we observed.

Furthermore, multivariate logistics analysis showed that, as well as longer duration of DM, lower eGFR was associated with the presence of plaque rupture. This is consistent with the study by Kakuta et al. [29], which demonstrated that renal function impacts the underlying pathophysiology of coronary plaque composition in patients with DM.

### Calcification

Hyperglycemia and DM promote vascular calcification via various mechanisms including production of advanced glycation end products, oxidative stress, and endothelial cell dysfunction [30]. The present study revealed the prevalence of calcification in culprit plaques to be higher among patients with DM than those without, consistent with a previous study that reported more frequent observation of superficial calcium and higher levels of calcium in culprit vessels of ACS among patients with DM compared with those without DM [31].

### Macrophage infiltration

A previous study reported that culprit-plaque rupture and the presence of a necrotic core with macrophage infiltration are independent predictors of worse outcome in patients with ACS [32]. In line with the concept of induction of a proinflammatory status by DM-mediated metabolic abnormalities, previous histopathological and OCT studies have reported that coronary plaques from patients with DM have increased macrophage infiltration compared with those from patients without DM [5, 6, 33]. Our study found the prevalence of macrophage infiltration to be higher in patients with DM than those without; however, the difference was not significant (P = 0.206). This discrepancy might be partly attributable to the relatively small sample size and selection of our study.

### Limitations

This study has several limitations which should be acknowledged. First, it is a single-center, retrospective study with a small sample size and highly selected population. Therefore, selection bias cannot be excluded. Second, although OCT enables reliable and effective qualification and quantification of coronary-plaque features, the findings still need to be validated in a wider population. Third, although we excluded cases with massive thrombus after sufficient thrombus aspiration, the underlying plaque morphology of the culprit lesion may have been obscured by residual thrombi in some of the enrolled patients. Finally, it is impossible to precisely determine the time period from onset of diabetes to cardiac attack. Therefore, we defined the duration of DM as the time from the first diagnosis with diabetes or from the first claimed prescription of glucose-lowering agents, to the time of STEMI attack.

## Conclusions

Our study provides evidence linking diabetes status with vulnerable features of coronary plaques in patients with AMI. In addition, we demonstrate that increased duration of DM combined with high HbA1c level significantly influences culprit-plaque characteristics in patients with DM who suffer AMI. These findings might account for the higher risks of cardiac death in DM patients with long disease duration, and inform clinical decision making or management of such patients, with consequent improvements in clinical outcomes.

## Supplementary information

**Additional file 1: Table S1.** Comparisons of characteristics of included and excluded patients

## Data Availability

The datasets used and/or analyzed during the current study are available from the corresponding author on reasonable request.

## Abbreviations

ACS: acute coronary syndrome
AMI: acute myocardial infarction
CAD: coronary artery disease
DM: diabetes mellitus
BMI: body mass index
FCT: fibrous cap thickness
HbA1c: glycated hemoglobin A1c
LDL-C: low-density-lipoprotein cholesterol
Long-DM: long duration DM group
MLA: minimal lumen area
Non-DM: patients without DM
OCT: optical coherence tomography
Short-DM: short duration DM group
TC: total cholesterol
TCFA: thin-cap fibroatheroma
T2DM: type 2 diabetes mellitus
